# Validation of the shotgun metabarcoding approach for comprehensively identifying herbal products containing plant, fungal, and animal ingredients

**DOI:** 10.1371/journal.pone.0286069

**Published:** 2023-07-03

**Authors:** Zhaolei Zhang, Weishan Mu, Weijun Kong, Jiali Liu, Jingyi Zhao, Qing Zhao, Mengmeng Shi, Hongye Zhao, Jinxin Liu, Linchun Shi

**Affiliations:** 1 Hebei Key Laboratory of Study and Exploitation of Chinese Medicine, Chengde Medical University, Chengde, China; 2 Institute of Medicinal Plant Development, Chinese Academy of Medical Sciences, Peking Union Medical College, Beijing, China; University of Palermo, ITALY

## Abstract

Identifying plant, fungal, and animal ingredients in a specific mixture remains challenging during the limitation of PCR amplification and low specificity of traditional methods. Genomic DNA was extracted from mock and pharmaceutical samples. Four type of DNA barcodes were generated from shotgun sequencing dataset with the help of a local bioinformatic pipeline. Taxa of each barcode was assigned by blast to TCM-BOL, BOLD, and GenBank. Traditional methods including microscopy, thin layer chromatography (TLC), and high-performance liquid chromatography (HPLC) were carried out according to Chinese pharmacopoeia. On average, 6.8 Gb shotgun reads were sequenced from genomic DNA of each sample. Then, 97, 11, 10, 14, and one operational taxonomic unit (OTU) were generated for *ITS2*, *psbA-trnH*, *rbcL*, *matK*, and *COI*, respectively. All the labeled ingredients including eight plant, one fungal, and one animal species were successfully detected in both the mock and pharmaceutical samples, in which Chebulae Fructus, Poria, and Fritilariae Thunbergia Bulbus were identified via mapping reads to organelle genomes. In addition, four unlabeled plant species were detected from pharmaceutical samples, while 30 genera of fungi, such as *Schwanniomyces*, *Diaporthe*, Fusarium were detected from mock and pharmaceutical samples. Furthermore, the microscopic, TLC, and HPLC analysis were all in accordance with the standards stipulated by Chinese Pharmacopoeia. This study indicated that shotgun metabarcoding could simultaneously identified plant, fungal, and animal ingredients in herbal products, which has the ability to serve as a valuable complement to traditional methods.

## Introduction

Identifying plant, fungal, and animal ingredients in a specific mixture remains challenging during the limitation of PCR amplification and low specificity of traditional methods. A combination of DNA metabarcoding, DNA barcoding, and high-throughput sequencing technology has been widely used in ecology [1–3], food science [4, 5], and other fields since first proposed in 2012 [6]. However, the polymerase chain reaction (PCR) amplification process is prone to bias and chimera generation, affecting subsequent data analysis [7]. To eliminate the influence of PCR bias or failure in some taxon groups, Liu et al. proposed a new strategy known as "shotgun metabarcoding," which directly enriched and assembled multiple DNA barcodes via shotgun sequencing data [8]. Shotgun metabarcoding successfully detected all the prescribed ingredients of Wuhu San in mock samples, while the feasibility of this method was verified by testing commercially available samples. Furthermore, shotgun metabarcoding was applied for the quality control of Qingguo Wan, where 103, 12, 10, and 12 OTUs were obtained for *ITS2*, *psbA-trnH*, *matK*, and *rbcL*, respectively, in mock and commercial samples [9]. All eight medicinal materials of Qingguo Wan were successfully authenticated, while fungi belonging to 20 genera in 19 families were detected in addition to the labeled ingredients. Moreover, 52.16 Gb of shotgun sequencing reads were generated for Fuke Desheng Wan, while 228, 23, and 14 operational taxonomic units (OTUs) were obtained for *ITS2*, *matK*, and *rbcL*, respectively [10]. Six labeled ingredients from the Apiaceae, Paeoniaceae, Asteraceae and Lamiaceae families were simultaneously detected, while 25 weed species representing 16 genera in ten families were revealed by the high-throughput sequencing data. Shotgun metabarcoding displays the potential to be used as a genetic identification method to simultaneously authenticate labeled ingredients, contaminants, and substitutes in traditional patented medicines.

Tiedi Wan, a classic prescription for treating chronic pharyngitis with a long history, was first recorded in the Ming Dynasty under "throat paralysis" in Volume 6 of Dr. Gong Tingxian’s "Shoushi Baoyuan"(寿世保元) [11]. According to the Pharmacopoeia of the People’s Republic of China (simplified as Chinese Pharmacopoeia, 2020 edition), Tiedi Wan consists of eight plant, one fungal, and one animal ingredient, i.e., Ophiopogonis Radix (Maidong), Platycodonis Radix (Jiegeng), Canarii Fructus (Qingguo), Scrophulariae Radix (Xuanshen), Fritilariae Thunbergia Bulbus (Zhebeimu), Trichosanthis Pericarpium (Gualoupi), Poria (Fuling), Glycyrrhiza Radix et Rhizoma (Gancao), Membrana Follicularis Ovi (Fenghuangyi), and Chebulae Fructus (Hezirou) [12]. The quality control methods of Tiedi Wan listed in the Chinese Pharmacopoeia inc lude the microscopic identification of the seven ingredients, thin layer chromatography (TLC) authentication of gallic acid and Peimine, and the content determination of glycyrrhizic acid via high-performance liquid chromatography (HPLC) [12]. In addition, glycyrrhetinic acid [13], platycodon saponin D, beriberiin A, and beriberiin B are reportedly quality markers of Tiedi Wan [14]. In another development, a novel electro-chemical detector was used for the detection of chemical components in Chinese medicine [15]. Compared to traditional methods, electrochemical sensors may be another superior option due to their low cost, high sensitivity and selectivity.

This study combined shotgun metabarcoding with microscopy, TLC, and HPLC to authenticate the biological ingredients in Tiedi Wan. Mock samples were prepared according to the 2020 edition of the Chinese Pharmacopoeia and used to test the feasibility of the method for identifying the labeled ingredients of Tiedi Wan. Then, commercially available pharmaceutical samples of Tiedi Wan were collected to simulate the process of quality detection to verify whether shotgun metabarcoding can be used as a complement to the traditional method.

## Materials and methods

### Herbal materials, mock samples, and pharmaceutical samples

**Fig 1:**
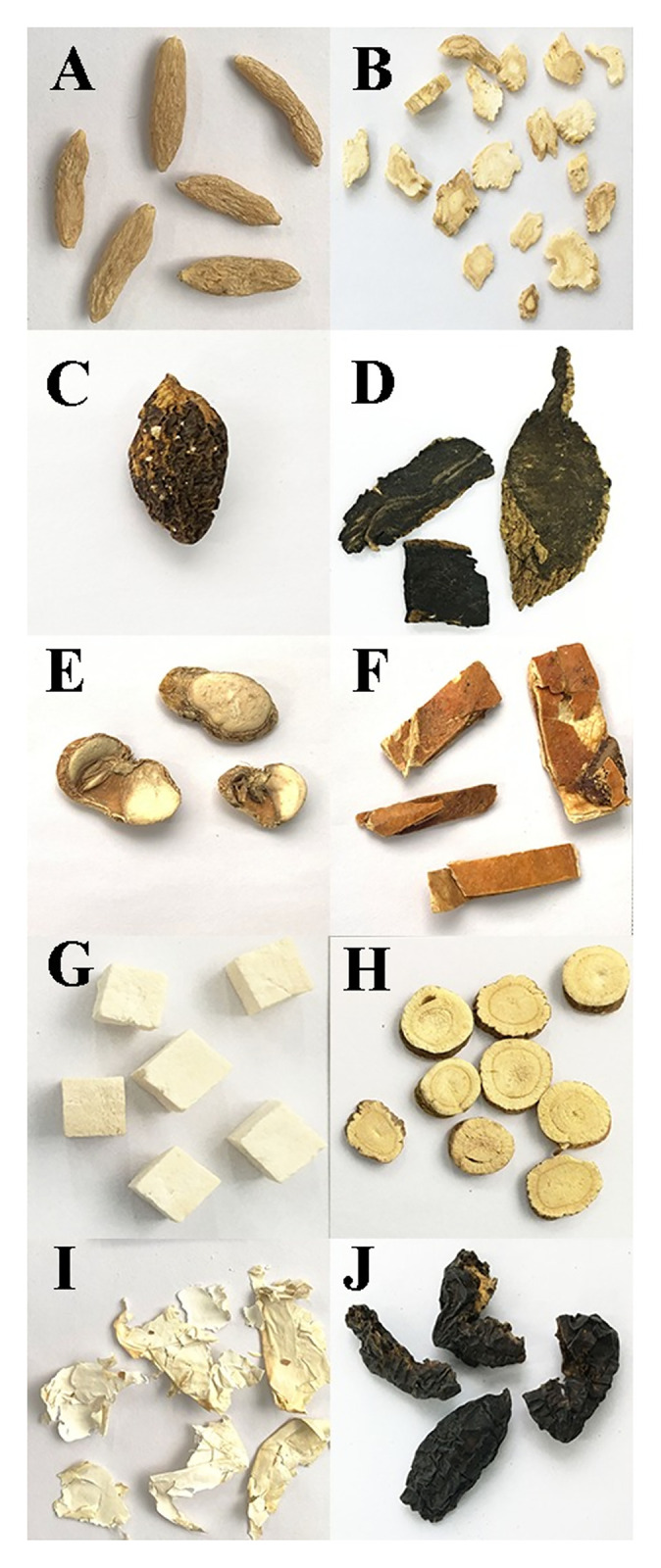
Morphological characteristics of the ten herbal materials of Tiedi Wan. (A) Ophiopogonis Radix, (B) Platycodonis Radix, (C) Canarii Fructus, (D) Scrophulariae Radix, (E) Fritilariae Thunbergia Bulbus, (F) Trichosanthis Pericarpium, (G) Poria, (H) Glycyrrhiza Radix Et Rhizoma, (I) Membrana Follicularis Ovi, (J) Chebulae Fructus.

The 10 medicinal materials (Fig 1) for preparing the mock samples were purchased from Tongrentang Pharmacy. The authenticity of these medicinal materials was verified via morphological and DNA barcoding technology. Two mock samples, namely HSZY162 and HSZY175, were prepared according to the prescriptions delineated in the Chinese Pharmacopoeia (S1 Table). The formulae for HSZY162 and HSZY175 were exactly the same except for Panacis Quinquefolii Radix, which was in the same proportion as Poria and was added to HSZY175 as a positive control for monitoring the experimental process (S2 Table). In addition, three batches of pharmaceutical samples were collected from the same manufacturer to simulate the behavior during market detection. The three batches of samples were labeled HSZY056, HSZY143, and HSZY144, respectively.

### DNA extraction, PCR amplification, Sanger sequencing, and Illumina sequencing

The DNA of the herbal materials and medicinal products was extracted according to previously reported methods by Liu et al [8, 9, 16]. The DNA quality of the herbal materials was estimated using a NanoDrop one ultra-micro spectrophotometer (Thermo Fisher Scientific Inc., USA). The DNA of the medicinal products was specifically quantified using a Qubit 4.0 Fluorometer (Thermo Fisher Scientific Inc., USA), followed by shearing to prepare a PCR-free library of 350 bp. The five traditional DNA barcoding regions, namely *ITS2*, *psbA-trnH*, *rbcL*, *matK*, and *COI*, were amplified using primer sets, ITS2F/ITS3R [17], *psbA/trnH* [18], 1F/724R [19], 3F_LIM/1R_KIM [20], and LCO1490/HCO2198 [21], and then bi-directionally sequenced using an ABI 3730 XL DNA Analyzer (Thermo Fisher Scientific Inc., USA). High throughput sequencing was conducted using the Illumina NovaSeq 6000 System.

### Data analysis

The *ITS2*, *psbA-trnH*, *matK*, *rbcL* and *COI*, primers were removed using CodonCode Aligner v9.0.1 to obtain the traditional DNA barcoding regions. All the DNA barcoding sequences were submitted and deposited in the GenBank database.

Adapter sequences and low-quality bases were gently clipped using Trimmomatic v0.38 [22]. The paired-end reads belonging to the *ITS2*, *psbA-trnH*, *matK*, *rbcL*, and *COI*, sequences were enriched via comparison with the GenBank public database. The enriched reads were assembled using MEGAHIT v1.2.9 and MetaSPAdes v3.13.0 [23, 24]. The DNA barcoding regions of *psbA-trnH*, *matK*, *rbcL* and *COI*, were obtained by removing the above primer sequences using cutadapt v3.8 [25], while the *ITS2* regions were annotated using the hidden Markov model (HMM)-based method [26]. The OTUs of the DNA barcodes were generated using Usearch v11 (https://www.drive5.com/usearch/) based upon a 100% sequence similarity threshold, while the representative sequence for each OTU was selected for further analysis. Next, the shotgun reads were mapped to each OTU using bowtie2 v2.3.4.3 [27] to calculate the OTU sequencing depths and coverage. Low reliable OTUs were removed according to previously reported thresholds [8]. The taxonomic assignment for each OTU was performed using the basic local alignment search tool (BLAST) according to the DNA barcoding system for traditional Chinese medicines (TCM-BOL), the barcode of life data system (BOLD), and GenBank on the basis of the lowest common ancestor (LCA) strategies provided by MEGAN v6.20.12 [28].

### Chemical analysis

For confirmation, the chemical tests for the pharmaceutical samples, including microscopic identification, TLC, and HPLC, were conducted according to the Chinese Pharmacopoeia. The pharmaceutical Tiedi Wan sample powder was observed under a biological microscope. Gallic acid and Peimine were used as thin-layer chromatographic identification controls to detect the presence of these two components in three commercially available samples according to the method delineated by the Chinese Pharmacopoeia. The TLC chromatograms of the sample and control show spots of the same color at corresponding positions. The glycyrrhetinic acid content was used as a test standard during HPLC. The chromatographic system for HPLC detection was as follows: an Agilent C18 chromatographic column (4.6 × 250 mm, 5 μm) was used as the stationary phase, while a mixture of methanol (A) and 0.2 mol/L Ammonium acetate solution (B) (64:36) was used as the mobile phase. The flow rate of the mobile phase was 1 ml/min, and the column temperature was set to 35 °C. The detector consisted of a diode array detector (DAD), and the spectrophotometer was set to 250 nm. The injection volume was 10 μl.

## Results

### Authentication of the medicinal materials for creating the mock samples using DNA barcoding and macroscopic identification methods

In addition to traditional macroscopic identification methods, DNA barcoding was used for the species confirmation of the ten ingredients. Six, seven, three, and five *ITS2*, *psbA-trnH*, *matK*, and *rbcL* barcodes were successfully obtained for the plant and fungal ingredients using universal primers, while no DNA barcodes were acquired for Chebulae Fructus and Poria. The COI, barcodes were obtained for the animal ingredient, Membrana Follicularis Ovi, verifying it as *Gallus gallus domesticus*. The *ITS2*, *psbA-trnH*, *matK*, and *rbcL* barcodes of the positive control, Panacis Quinquefolii Radix, have also been amplified and confirmed as *Panax quinquefolius*. In addition, the corresponding DNA barcodes of failed PCR were downloaded from previously published papers [16, 17]. The GenBank accession numbers of DNA barcodes obtained in this study and downloaded from GenBank are listed in Table 1.

**Table 1:** GenBank accession numbers of the medicinal materials for making mock samples.

Medicinal material	*ITS2*	*psbA-trnH*	*matK*	*rbcL*	*COI*
Ophiopogonis Radix	MZ491210	MZ540180	MZ556836	MZ540196	/
Platycodonis Radix	MZ491211	MZ540181	MZ556837	MZ540197	/
Canarii Fructus	MZ491216	MZ540187	KJ510921^1^	MZ540201	/
Scrophulariae Radix	MZ491213	MZ540182	NC053823^1^	GQ436721^1^	/
Fritilariae Thunbergia Bulbus	MF096328^1^	MZ540183	JQ724626^1^	KF850894^1^	/
Trichosanthis Pericarpium	MZ491218	MZ540189	MZ556839	MZ540203	/
Poria	EF397597^1^	/	/	/	/
Glycyrrhiza Radix et	MZ491219	MZ540190	AB280741^1^	MZ540204	/
Membrana Follicularis Ovi	/	/	/	/	MZ540207
Chebulae Fructus	MF096796^1^	LC435437^1^	MG737419^1^	KT203922^1^	/
Panacis Quinquefolii Radix	MT102865	MT994329	MW000341	MW000333	/

NOTE: The "1" symbol in the top right corner of the accession numbers indicates that it was downloaded from GenBank. The "/" indicates that the locus is not present in the genome of the medicinal material.

### Summary of the shotgun meta-sequencing and data analysis

A total of 34.08 Gb of clean data were generated for the five samples via high-throughput sequencing using the Illumina NovaSeq platform, yielding 101,861, 754,010, 9,317, 16,322, and 8,113 paired-end reads for *ITS2*, *psbA-trnH*, *matK*, *rbcL*, and *COI*, respectively. Furthermore, 11,392 unique contigs were generated via the MEGAHIT and MetaSPAdes assemblers. Annotation of the DNA barcoding regions yielded 243, 35, 26, 28, and 2 *ITS2*, *psbA-trnH*, *matK*, *rbcL*, and *COI*, barcodes, respectively. Finally, 133 high-quality OTUs were obtained with the similarity of 100%. The average length of *ITS2*, *psbA-trnH*, *matK*, *rbcL*, and *COI*, were 203bp, 374bp, 881bp, 702bp, and 658 bp. A summary of the shotgun meta-sequencing and data analysis results can be found in Table 2 and S3 Table.

**Table 2:** Summary of the data analysis of shotgun meta-sequencing data.

	*ITS2*	*psbA-trnH*	*matK*	*rbcL*	*COI*
Number of unique contig	598	10458	48	126	162
Number of barcordes after annotation chimera	243	35	26	28	2
Number of OTU	97	11	10	14	1
Average length (bp)	203.4	373.6	881.1	702.2	658
GC content (%)	57.9	31.5	32.8	42.8	49.7

### The DNA barcodes of the medicinal materials detected in the mock samples

Here, 15, 9, 8, and 10 OTUs were generated for *ITS2*, *psbA-trnH*, *matK*, and *rbcL* in the two mock samples (HSZY162 and HSZY174) created in the laboratory. The species assignment of these OTUs showed that the shotgun meta-sequencing approach detected all the prescribed ingredients except Chebulae Fructus and Poria (Table 3).

**Table 3:** High throughput sequencing (HTS) detection of the DNA barcode sequences of prescribed herbal materials in two TDW samples.

Species	HSYZ162					HSYZ174				
	*ITS2*	*psbA-trnH*	*matK*	*rbcL*	*COI*	*ITS2*	*psbA-trnH*	*matK*	*rbcL*	*COI*
*Ophiopogon japonicus*	-	√	√	√	/	√	√	√	√	/
*Platycodon grandiflorus*	√	√	√	√	/	√	√	√	√	/
*Canarium album*	√	√	√	√	/	√	√	√	√	/
*Scrophularia ningpoensis*	√	√	√	√	/	√	√	√	√	/
*Fritillaria thunbergii*	√	√	√	√	/	√	√	√	√	/
*Trichosanthes kirilowii*	√	√	√	√	/	√	√	√	√	/
*Wolfiporia cocos*	-	/	/	/	/	-	/	/	/	/
*Glycyrrhiza uralensis*	√	√	√	√	/	√	√	√	√	/
*Gallus gallus*	/	/	/	/	√	/	/	/	/	√
*Terminalia chebula*	-	-	-	-	/	-	-	-	-	/

NOTE: "√" indicates that the corresponding DNA barcode of this species was obtained, and "- " indicates that the corresponding DNA barcode of this species cannot be obtained, and "/" indicates that this herbal material was not added to the sample.

**Fig 2:**
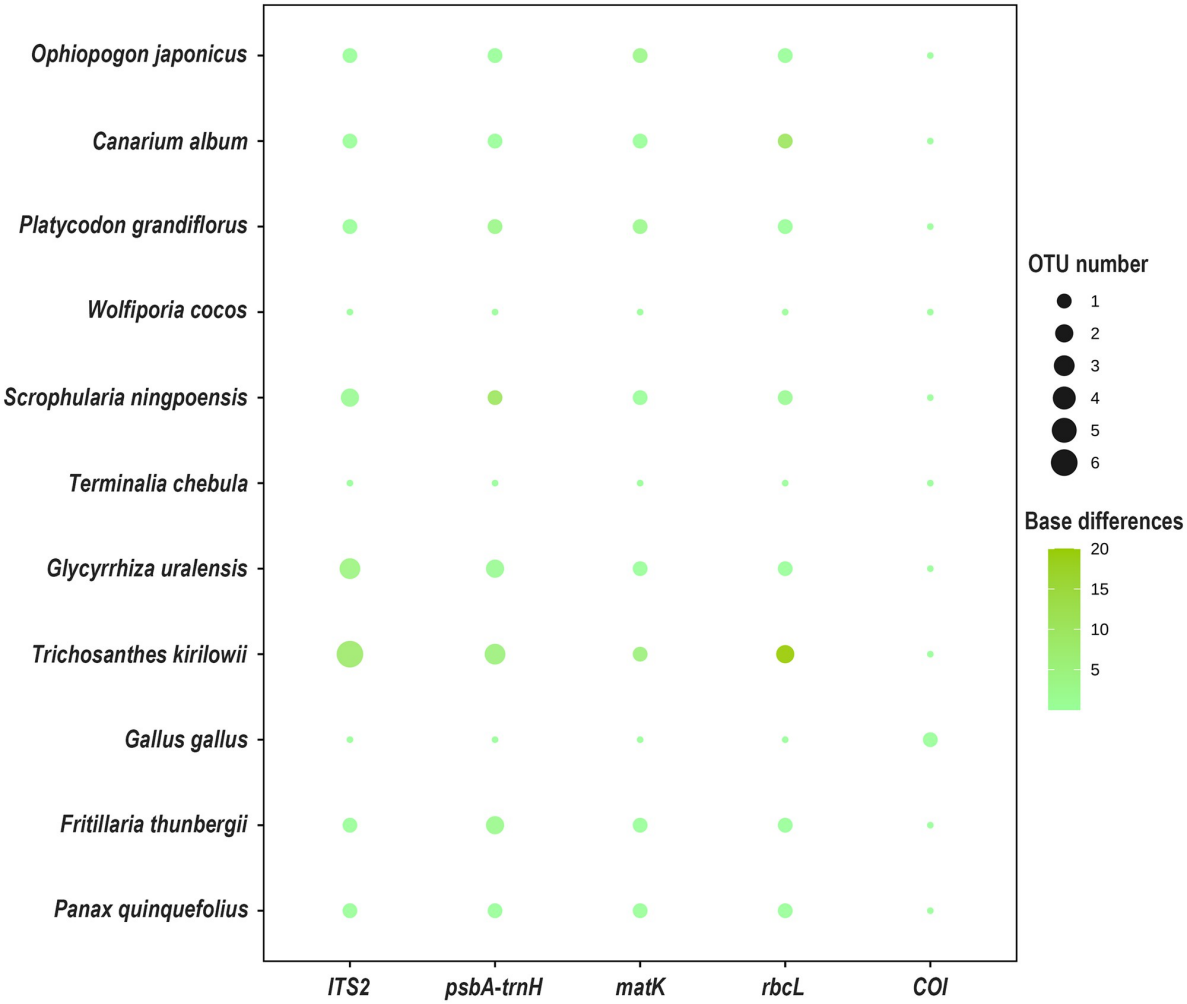
The number of OTUs and base differences detected in mock samples of Tiedi Wan groups of species. The size of the bubbles indicates the number of OTUs obtained and the colour of the bubbles indicates base differences. Darker colours indicate more base differences.

The *ITS2* sequences of Glycyrrhiza Radix et Rhizoma in the *ITS2* region displayed three OTUs, while Scrophulariae Radix had three, and Trichosanthis Pericarpium had six. The identification of OTUs showed that the ITS2 sequence of Scrophulariae Radix clustered 2 OTUs, 1 of which differed by 1 base from the reference sequence obtained in Section 3.1 using the Sanger sequencing method. Three OTUs were obtained for Glycyrrhiza Radix et, with 0–4 base differences compared to the reference sequence. The ITS2 sequence of Trichosanthis Pericarpium had 6 OTUs, of which 1 OTU was consistent with the reference sequence and the other 5 OTUs differed from the reference sequence by 8 base sites. All the other prescribed ingredients obtained one type of OTU, respectively, which was the same as the sequences generated via Sanger sequencing. For the *psbA-trnH*, *matK*, and *rbcL* barcodes, the OTUs obtained via shotgun meta-sequencing were identical or exhibited a one-base difference compared with the sequences of corresponding species obtained via Sanger sequencing. Only one type of COI, OTU was clustered in the two mock samples, while the species were identified as *Gallus gallus domesticus*. In addition, the four barcodes of Panacis Quinquefolii Radix, as a positive control for the experiment, were all successfully re-assembled and exactly the same as the sequences obtained in Section 3.1. An overview of the DNA barcodes belonging to the prescribed ingredients is shown in Fig 2.

### The plant and animal ingredients detected in the commercial pharmaceutical samples

**Fig 3:**
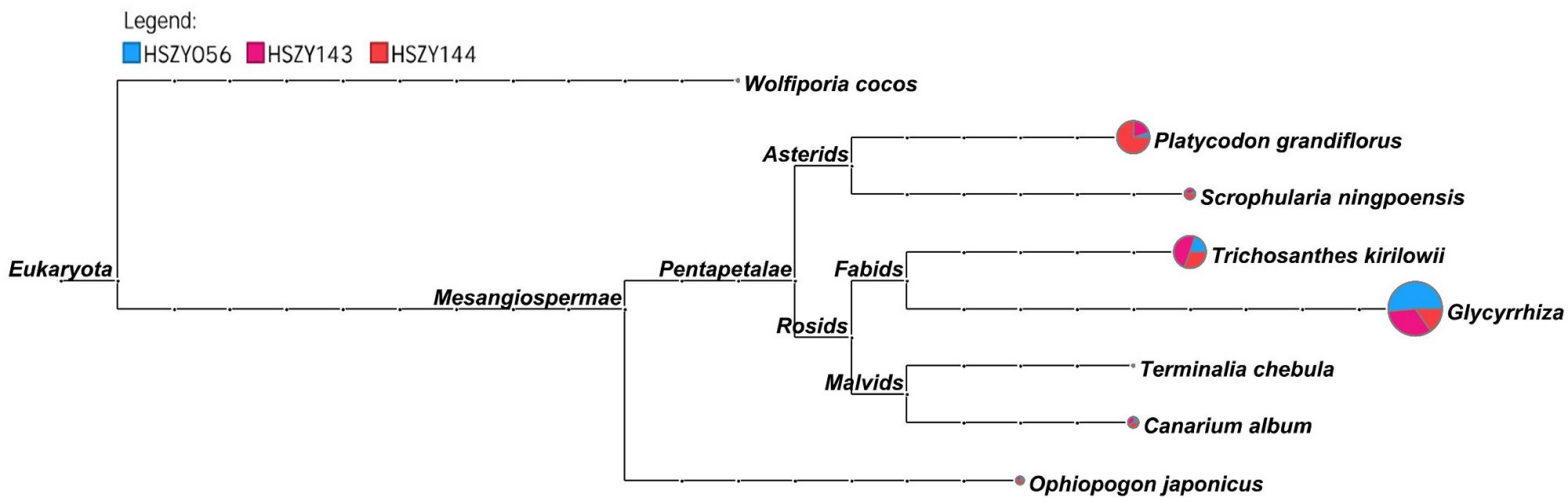
The *ITS2* sequences of three commercially available samples were clustered and analysed using MEGAN6. Each taxonomic the node is drawn as a pie chart indicating the proportion of each species in the taxon for each sample.

The labeled plant ingredients in the prescription, as well as the *ITS2*, *psbA-trnH*, *matK*, and *rbcL* barcodes, were all successfully detected in the pharmaceutical samples. *ITS2* sequence detection identified the six labeled ingredients in all the samples, while Chebulae Fructus and Poria were detected as partial sequences in one or two samples, and none of the samples contained the *ITS2* sequence for Fritilariae Thunbergia Bulbus (Fig 3, S4 Table). For the *psbA-trnH* and *rbcL* barcodes, Trichosanthis Pericarpium, Scrophulariae Radix, and Glycyrrhiza Radix et Rhizoma were detected in all pharmaceutical samples (S1 and S2 Figs, S5 and S6 Tables). Moreover, the *matK* barcodes of Canarii Fructus, Platycodonis Radix, Trichosanthis Pericarpium, and Glycyrrhiza Radix et Rhizoma were present in all the pharmaceutical samples (S3 Fig, S7 Table). In addition, shotgun sequencing also detected several plant source ingredients not labeled in the prescription, such as Cullen corylifolium, Limonium gmelinii, Lablab purpureus, Toona sinensis, Alhagi sparsifolia and Zea mays, while the number of reads belonging to the unlabeled plant ingredients was 1.37% that of the labeled plant ingredients. Finally, one type of COI, sequence was detected in all the pharmaceutical samples, which was identified as Gallus gallus domesticus, referring to the legal original species of Membrana Follicularis Ovi.

### Organelle genome mapping for Poria and Chebulae Fructus that cannot be stably detected

In this study, the completed DNA barcodes of Poria and Chebulae Fructus could only be detected in HSZY144 and HSZY056. By mapping their *ITS2* sequences, only partial Poria and Chebulae Fructus sequences could be obtained, while a few reads were mapped in other samples.

When mapping reads to the whole mitochondrial genome of Poria, the *nad1*, *orf5*, *nad2*, *COX1*, *atp8*, and orf30 regions of the mitochondrial genome were mapped by reads in HSZY056 and HZSZY143, while only the orf5, and *COX1* regions were covered in HSZY162 and HSZY174. The *COX1* region was mapped by 20 reads in sample HSZY162, while most of the reads were distributed in the region between primer LCO1490 and HCO2198 (S4 Fig). A fewer number of reads were evident in the *orf5* region than in the *COX1* region and were similar in all four samples.

For the whole chloroplast genome of Chebulae Fructus, 80478, 11840, 138276, 2294458 paired-end reads were mapped in 27 genes and 18 intergenic regions in HSZY143, HSZY144, HSZY162 and HSZY174, respectively. The *psbA*, *ndhK*, *ndhB*, *ycf2*, and *rpl2* genes could be mapped with reads in all the samples. The *psbA*, *ndhK*, *ndhB*, *ycf2*, and *rpl2* gene was mapped with relatively more reads in the four samples, and the *psbA* and *rpl2* regions had higher coverage and sequencing depth in these gene. The *psbA* and *rpl2* regions were mapped by much more reads than other regions in the four sample, with an average coverage of 99.43% and 99.30% respectively. The *psbA* region of Chebulae Fructus in HSZY162 alone displayed 100% coverage with 1542 reads (S5 Fig). More reads were mapped on the intergenic gene spacers than genes such as *rpl36-rps8*, *rps12-ndhF*, and *ycf1-rps12*.

### The fungi detected in the mock and pharmaceutical samples

Fifty-five *ITS2* OTUs were identified as fungal belonging to 30 genera of 22 families (S6 Fig, S8 Table). The number of fungal *ITS2* reads was 1.62% that of the total *ITS2* reads. *Schwanniomyces* was the most dominant in all the mock and pharmaceutical samples, followed by *Diaporthe*, *Fusarium*, *Penicillium*, and *Aspergillus*. The number of *Schwanniomyces* reads was 21.5% of the total number of reads belonging to the fungal community. *Aspergillus* was detected in both the mock and pharmaceutical samples, with a total of 250 reads. The number of *Aspergillus* reads in the pharmaceutical samples was 202, which was about four times that of the mock samples. In addition, the number of fungal genera detected in each sample differed, with a minimum of three in HSZY162 and a maximum of 20 in HSZY144. The prescription ingredient Poria is a fungus, of which 46 reads were detected. The *ITS2* sequence of Poria was assembled into a complete DNA barcode in HSZY144 and into partial sequences in HSZY056 and HSZY143.

### Microscopic identification and chemical analysis of the pharmaceutical samples

**Fig 4:**
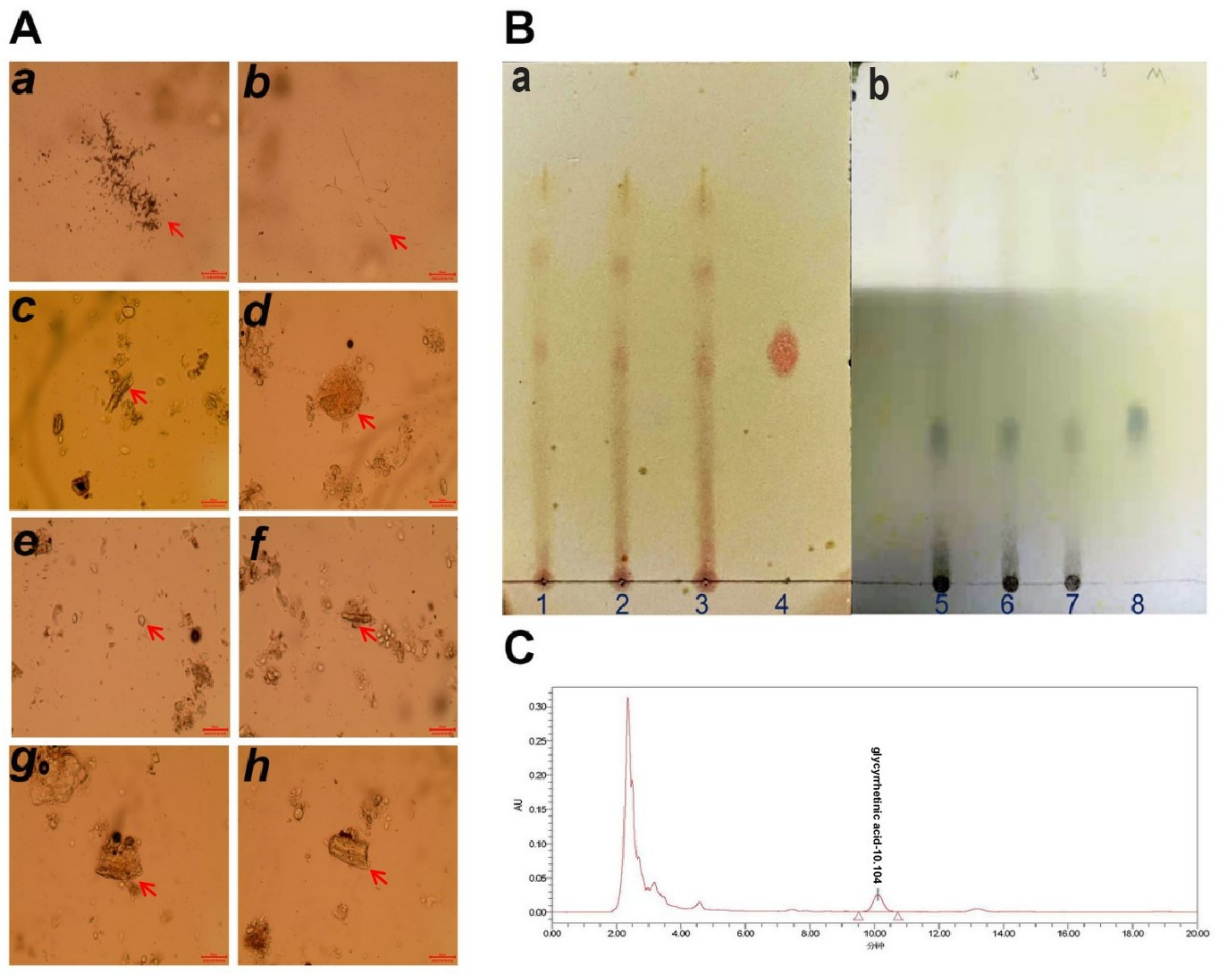
Microscopic identification, the TLC identification, and HPLC chromatogram of pharmaceutical samples. A. Microscopical identification: a. Irregularly branched mass b. hypha c. Calcium oxalate needle crystal d. stone cell e. amylum body f. Fibrous layer of pericarp g. Connecting tube h. Crystal Sheath Fiber. B. Figure a shows the thin layer chromatogram of gallic acid, 1–3 represent the test solutions of HSZY056, HSZY143 and HSZY144 respectively, and 4 represents the control solution of gallic acid. Figure b shows the thin layer chromatogram of Peimine. 5–7 represent the sample solutions of HSZY056, HSZY143 and HSZY144 respectively, and 8 is the control solution of Peimine. C. HPLC chromatogram of pharmaceutical samples. The peak marked in the figure is the chromatographic peak of glycyrrhizic acid.

Analysis of the sample powders revealed the microscopic features of seven species, including Poria, Ophiopogonis Radix, Scrophulariae Radix, Fritilariae Thunbergia Bulbus, Chebulae Fructus, Platycodonis Radix, and Glycyrrhiza Radix et Rhizoma (Fig 4A). The microscopic features of Poria showed irregularly branched masses and a light brown mycelium. Ophiopogonis Radix presented bundled or scattered calcium oxalate needles of 24–50 μm long and about 3 μm in diameter. Scrophulariae Radix were yellowish-brown stone cells, which were round or irregularly shaped and approximately 94 μm in diameter. Fritilariae Thunbergia Bulbus displayed an ovoid amylum body. The microscopic features of Chebulae Fructus of the chebulo are the yellowish fibrous layer of pericarp. Platycodonis Radix displayed tubes of 14–25 μm in diameter, containing yellowish granular material. Glycyrrhiza Radix et Rhizoma exhibited crystal fibers formed via calcium oxalate square crystals contained in the thin-walled cells surrounding the fiber bundles. The TLC chromatograms of the Tiedi Wan samples with gallic acid and Peimine are shown in Fig 4B. The points in the chromatograms obtained for the test solutions of the three Tiedi Wan samples correspond to the position and color of the spots acquired for the control solution. The three pharmaceutical samples contained 3.06 mg/g, 3.09 mg/g, and 3.05 mg/g of glycyrrhetinic acid as the active ingredient and were detected via HPLC (Fig 4C). The results of the three pharmaceutical samples all met the standards of the Chinese Pharmacopoeia.

## Discussion

### The challenges of using shotgun metabarcoding to characterize the species in traditional herbal products

The analytical results of the mock samples showed four DNA barcode sequences for most of the herbs. The high throughput data of HSZY174 revealed the presence of the positive control, Panacis Quinquefolii Radix, indicating that this method could be used to detect species with a low dosage and high sensitivity. However, the initial study showed two OTUs in the *ITS2* fragment for Ginseng Radix et Rhizoma and Panacis Quinquefolii Radix. A comparison with the standard sequences of Ginseng Radix et Rhizoma and Panacis Quinquefolii Radix revealed that these two OTUs were located at positions 34 and 43, which belonged to the Ginseng Radix et Rhizoma and Panacis Quinquefolii Radix sequences, respectively. The original data was used to map the Panacis Quinquefolii Radix OTU, indicating that the Ginseng Radix et Rhizoma sequence was absent from this data, probably due to an assembly error generated by the assembly software.

The *ITS2* region showed an abundance of Glycyrrhiza Radix et Rhizoma, Scrophulariae Radix, and Trichosanthis Pericarpium OTUs with base differences from the DNA barcodes obtained via Sanger sequencing in Section 3.1. The OTU differences obtained via the two sequencing methods may be related to the *ITS2* region being a duplicate gene. High-throughput sequencing obtains the genome of a mixture of multiple individuals, with sequences that may be present in multiple cases. Contrarily, Sanger sequencing can only obtain one gene sequence by testing a sample segment at a time.

The *ITS2* sequence of Ophiopogonis Radix was detected in HSZY174 but not in HSZY162. After assembly using the original data, some Ophiopogonis Radix *ITS2* sequences were also found in HSZY162, probably due to uneven sequencing. The chloroplast genes of Ophiopogonis Radix and Canarii Fructus were not detected in the HSZY143, and HSZY144 pharmaceutical samples, probably since the chloroplast gene sequencing was not uniform and the sequences of these three DNA barcode regions were not obtained. Four barcode sequences were detected in both mock samples but not in the pharmaceutical samples.

Northern genistein was detected during the sequencing data analysis. Scrophulariae Radix is the dried root of the plant *Scrophularia ningpoensis* Hemsl, which belongs to the genus *Scrophularia* in the Scrophulariaceae family. There are two species, *S*. *ningpoensis* and *S*. *buergeriana* (*S*. *oldhami*), both of which are similar in form and differ only in inflorescence and flower color [29]. In 1959, *S*. *ningpoensis* was used for the first time as the base of Scrophulariae Radix in the first edition of Chinese Medicine and was also known as Zhejiang Scrophulariae Radix. Due to its abundance and good quality, *S*. *ningpoensis* has been used as the base of Scrophulariae Radix in all editions of the Pharmacopoeia. In the northern part of China, *S*. *ningpoensis* is often used as a substitute for *S*. *buergeriana* since they are equal in anti-inflammatory potency and analgesic effect. Therefore, it is feasible to use *S*. *ningpoensis* as a substitute for *S*. *buergeriana* in clinical practice [30].

Some non-prescription ingredients and fungi were detected in the pharmaceutical samples via *ITS2* sequences with a small proportion of reads. In previous tests, sequences of non-prescribed ingredients and fungi were obtained in all analyses of proprietary Chinese medicines by Liu et al. Salix sp., Chenopodium album, Convolvulus arvensis, Citrus sp., and Scutellaria baicalensis were detected as non-prescription ingredients and 24 fungal genera in Wuhu San [8]. Fungal components of 19 families and 20 genera were detected during a study of the Qingguo Wan [9]. A total of 25 weed species and 26 fungal genera belonging to 17 families were detected in both lab-made and commercial Fuke Desheng Wan samples [10]. The detection of non-prescription ingredients may be due to the introduction of other impurities during the production and processing of Chinese medicines [31]. Fungi were detected in all samples, which may be introduced during the production, processing, transportation, and storage of traditional patent medicine [32]. Using a shotgun metabarcoding method to obtain DNA from all species in the sample for analysis, multiple species such as prescription ingredients, non-prescription ingredients and fungi are detected and species detection of mixtures can be accomplished. Shotgun metabarcoding is suitable for the identification of a wide range of unknown herbal raw materials or processed products.

### The complementarity of using multiple DNA barcodes for species detection in traditional herbal products

This study used five DNA barcodes to detect the components of traditional herbal medicine, namely, *ITS2*, *psbA-trnH*, *matK*, *rbcL*, and *COI*, fragments, with different identification abilities. The *ITS2* region can be used as a standard DNA barcode to identify medicinal plants and their relatives for determining a wider range of plant taxa [17, 33]. All the *ITS2* sequences obtained in this study were identified accurately and displayed higher identification efficiency compared to the sequences of other fragments.

Chloroplasts are present in green tissues and immature seeds in the form of protoplasts [34]. Chebulae Fructus is the dried ripe fruit of Terminalia chebula Retz. or Terminalia tomentella Retz. var. tomentella Kurt. of the family Angelicaceae and chloroplast genes are not easily detected. Chloroplast genes are absent in some herbal materials, such as fungal herbs like Poria, in which three chloroplast genes could not be detected. The commonly used single DNA barcodes cannot identify all species and need to be combined with other DNA barcodes for identification [35]. The three single-gene barcodes present their own advantages and disadvantages, with *matK* having a high evolutionary rate, moderate length, and obvious interspecific differentiation [36] but with different identification capabilities for different species [35]. The *rbcL* could be used to identify the level of family and genus, but not the species [37], while it could be used in combination with other barcodes for accurate identification [38]. The properties of *psbA-trnH* show that it was more suitable as a universal DNA barcode. Amplification primers were obtained in conserved coding regions. A single primer pair could amplify almost all angiosperms with large sequence variation in the spacer region [39, 40]. The mock sample results indicated that the three chloroplast genes could be used as auxiliary barcodes for identifying herbal components. The Ophiopogonis Radix sequences were not found in the HSZY056, HSZY143, and HSZY144 pharmaceutical samples. The whole chloroplast genome was used to assemble the raw data of these three samples. Although the *matK*, *rbcL*, and *psbA-trnH* sequences were not obtained, a portion of other chloroplast sequences was identified. Using the whole chloroplast genome as DNA barcodes solved the problem of insufficient variation in a single barcode. Species are distinguished by bioinformatics identification, combined with other methods to ensure material quality [41].

## Conclusions

The results suggest that the shotgun metabarcoding approach can authenticate all the biological ingredients of Tiedi Wan. This study confirms that shotgun metabarcoding displays potential as a powerful tool to simultaneously authenticate plant, fungal, and animal ingredients in Tiedi Wan, ensuring its quality in combination with other traditional methods.

## Supporting information

S1 Table Medicinal materials collected for making mock samples. (DOCX)

S2 Table The proportion and dosage of herbal materials listed in the prescription of mock and pharmaceutical drug Tiedi Wan according to the Chinese Pharmacopoeia. (DOCX)

S3 Table Sequencing results of the TDW samples. (DOCX)

S4 Table The reads number of the prescription ingredients in the three commercially available samples based on the *ITS2* sequences. (DOCX)

S5 Table The reads number of the prescription ingredients in the three commercially available samples based on the *psbA-trnH* sequences. (DOCX)

S6 Table The reads number of the prescription ingredients in the three commercially available samples based on the *rbcL* sequences. (DOCX)

S7 Table The reads number of the prescription ingredients in the three commercially available samples based on the *matK* sequences. (DOCX)

S8 Table The reads number of the fungi in the five samples based on the *ITS2* sequences. (DOCX)

S1 Fig The *psbA-trnH* sequences of three commercially available samples were clustered and analysed using MEGAN6. The node is drawn as a pie chart indicating the proportion of each species in the taxon for each sample.
(TIF)

S2 Fig The *rbcL* sequences of three commercially available samples were clustered and analysed using MEGAN6. Each taxonomic the node is drawn as a pie chart indicating the proportion of each species in the taxon for each sample.
(TIF)

S3 Fig The *matK* sequences of three commercially available samples were clustered and analysed using MEGAN6. Each taxonomic the node is drawn as a pie chart indicating the proportion of each species in the taxon for each sample.
(TIF)

S4 Fig Mapping of the *COX1* sequence of the mitochondrial genome of Poria in HSZY162. (TIF)

S5 Fig Mapping of the *psbA* sequence of the chloroplast genome of Chebulae Fructus in HSZY162. (TIF)

S6 Fig Distribution of the fungi for each sample at the genus level. The data were visualized by Circos. The left half-circle indicates the distribution ratio of species in different samples at the genus level: the outer ribbon represents the species; the inner ribbon represents different groups, and the length represents the sample proportion of a particular genus. The right half-circle indicates the species composition in each sample: the color of the outer ribbon represents samples from different groups; the color of the inner ribbon represents the composition of different species in each sample, and the length of the ribbon represents the relative abundance of the corresponding species.
(TIF)
