# Supplementary material for: Validation of the shotgun metabarcoding approach for comprehensively identifying herbal products containing plant, fungal, and animal ingredients
Source: PLoS One. 2023 Jul 3;18(7):e0286069. doi: 10.1371/journal.pone.0286069 (PMC10317219; doi:10.1371/journal.pone.0286069)
Supplement: S1 Table — (DOCX) [file pone.0286069.s001.docx]

**Supplementary Material**

## Supplementary Tables

**S1 Table. Medicinal materials collected for making mock samples.**

| Chinese name | medicinal material | Latin name/scientific name | Medicinal part |
| --- | --- | --- | --- |
| Maidong | Ophiopogonis Radix | *Ophiopogon japonicus* | Tuber |
| Jiegeng | Platycodonis Radix | *Platycodon grandiflorum* | Root |
| Qingguo | Canarii Fructus | *Canarium album* | Ripe fruit |
| Xuanshen | Scrophulariae Radix | *Scrophularia ningpoensis* | Root |
| Zhebeimu | Fritilariae Thunbergia Bulbus | *Fritillaria thunbergii* | Bulb |
| Gualoupi | Trichosanthis Pericarpium | *Trichosanthes kirilowii* | Ripe seed |
| Fuling | Poria | *Poria cocos* | Sclerotium |
| Gancao | Glycyrrhiza Radix Et Rhizoma | *Glycyrrhiza uralensis* | Root and rhizome |
| Fenghuangyi | Membrana Follicularis Ovi | *Gallus gallus domesticus* | Egg inner shell membrane |
| Hezirou | Chebulae Fructus | *Terminalia chebula* | Ripe fruit |
| Xiyangshen | Panacis Quinquefolii Radix | *Panax quinquefolius* | Root |
