# Supplementary material for: Validation of the shotgun metabarcoding approach for comprehensively identifying herbal products containing plant, fungal, and animal ingredients
Source: PLoS One. 2023 Jul 3;18(7):e0286069. doi: 10.1371/journal.pone.0286069 (PMC10317219; doi:10.1371/journal.pone.0286069)
Supplement: S2 Table — (DOCX) [file pone.0286069.s002.docx]

**Supplementary Material**

## Supplementary Tables

**S2 Table. The proportion and dosage of herbal materials listed in the prescription of mock and pharmaceutical drug Tiedi Wan according to the Chinese Pharmacopoeia.**

| Pinyin Name | medicinal material | Dosage(g) | Proportion (%) |
| --- | --- | --- | --- |
| Maidong | Ophiopogonis Radix | 150 | 8.69 |
| Jiegeng | Platycodonis Radix | 300 | 17.40 |
| Qingguo | Canarii Fructus | 60 | 3.48 |
| Xuanshen | Scrophulariae Radix | 150 | 8.69 |
| Zhebeimu | Fritilariae Thunbergia Bulbus | 300 | 17.40 |
| Gualoupi | Trichosanthis Pericarpium | 150 | 8.69 |
| Fuling | Poria | 150 | 8.69 |
| Gancao | Glycyrrhiza Radix et Rhizoma | 300 | 17.40 |
| Fenghuangyi | Membrana Follicularis Ovi | 15 | 0.87 |
| Hezirou | Chebulae Fructus | 150 | 8.69 |
