# Supplementary material for: Validation of the shotgun metabarcoding approach for comprehensively identifying herbal products containing plant, fungal, and animal ingredients
Source: PLoS One. 2023 Jul 3;18(7):e0286069. doi: 10.1371/journal.pone.0286069 (PMC10317219; doi:10.1371/journal.pone.0286069)
Supplement: S3 Table — (DOCX) [file pone.0286069.s003.docx]

**Supplementary Material**

## Supplementary Tables

**S3 Table. Sequencing results of the TDW samples.**

| Sample ID | Number of bases | Total reads | The number of each DNA barcode | | | | | |
| --- | --- | --- | --- | --- | --- | --- | --- | --- |
|  |  |  | *ITS2* | *psbA-trnH* | *matK* | *rbcL* | *COI* | Total |
| HSZY056 | 7391118600 | 24637062 | 14485 | 137156 | 1943 | 3134 | 1662 | 172206 |
| HSZY143 | 5570973000 | 18569910 | 9246 | 87594 | 1162 | 2105 | 1359 | 110233 |
| HSZY144 | 6446504700 | 21488349 | 13103 | 127627 | 1504 | 1753 | 1766 | 159418 |
| HSZY162 | 6570641400 | 21902138 | 21758 | 137275 | 1763 | 3018 | 1276 | 182885 |
| HSZY174 | 8095645500 | 26985485 | 43269 | 264358 | 2945 | 6312 | 2050 | 359456 |
