# Supplementary material for: Validation of the shotgun metabarcoding approach for comprehensively identifying herbal products containing plant, fungal, and animal ingredients
Source: PLoS One. 2023 Jul 3;18(7):e0286069. doi: 10.1371/journal.pone.0286069 (PMC10317219; doi:10.1371/journal.pone.0286069)
Supplement: S4 Table — (DOCX) [file pone.0286069.s004.docx]

**Supplementary Material**

## Supplementary Tables

**S4 Table. The reads number of the prescription ingredients in the three commercially available samples based on the *ITS2* sequences.**

| medicinal material | HSZY056 | HSZY143 | HSZY144 |
| --- | --- | --- | --- |
| Ophiopogonis Radix | 41 | 18 | 46 |
| Platycodonis Radix | 155 | 196 | 1030 |
| Canarii Fructus | 101 | 55 | 84 |
| Scrophulariae Radix | 38 | 40 | 82 |
| Fritilariae Thunbergia Bulbus | 0 | 0 | 0 |
| Trichosanthis Pericarpium | 600 | 449 | 380 |
| Poria | 0 | 0 | 31 |
| Glycyrrhiza Radix et Rhizoma | 4167 | 2956 | 3508 |
| Membrana Follicularis Ovi | 0 | 0 | 0 |
| Chebulae Fructus | 19 | 0 | 0 |
