# Supplementary material for: Validation of the shotgun metabarcoding approach for comprehensively identifying herbal products containing plant, fungal, and animal ingredients
Source: PLoS One. 2023 Jul 3;18(7):e0286069. doi: 10.1371/journal.pone.0286069 (PMC10317219; doi:10.1371/journal.pone.0286069)
Supplement: S6 Table — (DOCX) [file pone.0286069.s006.docx]

**Supplementary Material**

## Supplementary Tables

**S6 Table. The reads number of the prescription ingredients in the three commercially available samples based on the *rbcL* sequences.**

| medicinal material | HSZY056 | HSZY143 | HSZY144 |
| --- | --- | --- | --- |
| Ophiopogonis Radix | 0 | 0 | 0 |
| Platycodonis Radix | 0 | 0 | 27 |
| Canarii Fructus | 27 | 0 | 0 |
| Scrophulariae Radix | 53 | 0 | 52 |
| Fritilariae Thunbergia Bulbus | 0 | 0 | 0 |
| Trichosanthis Pericarpium | 31 | 50 | 48 |
| Poria | 0 | 0 | 0 |
| Glycyrrhiza Radix et Rhizoma | 1187 | 907 | 1031 |
| Membrana Follicularis Ovi | 0 | 0 | 0 |
| Chebulae Fructus | 0 | 0 | 0 |
