# Supplementary material for: Validation of the shotgun metabarcoding approach for comprehensively identifying herbal products containing plant, fungal, and animal ingredients
Source: PLoS One. 2023 Jul 3;18(7):e0286069. doi: 10.1371/journal.pone.0286069 (PMC10317219; doi:10.1371/journal.pone.0286069)
Supplement: S7 Table — (DOCX) [file pone.0286069.s007.docx]

**Supplementary Material**

## Supplementary Tables

**S7 Table. The reads number of the prescription ingredients in the three commercially available samples based on the *matK* sequences.**

| medicinal material | HSZY056 | HSZY143 | HSZY144 |
| --- | --- | --- | --- |
| Ophiopogonis Radix | 0 | 0 | 0 |
| Platycodonis Radix | 42 | 0 | 148 |
| Canarii Fructus | 75 | 14 | 23 |
| Scrophulariae Radix | 23 | 0 | 31 |
| Fritilariae Thunbergia Bulbus | 0 | 0 | 0 |
| Trichosanthis Pericarpium | 84 | 111 | 73 |
| Poria | 0 | 0 | 0 |
| Glycyrrhiza Radix et Rhizoma | 1296 | 776 | 862 |
| Membrana Follicularis Ovi | 0 | 0 | 0 |
| Chebulae Fructus | 0 | 0 | 0 |
