# Supplementary material for: Validation of the shotgun metabarcoding approach for comprehensively identifying herbal products containing plant, fungal, and animal ingredients
Source: PLoS One. 2023 Jul 3;18(7):e0286069. doi: 10.1371/journal.pone.0286069 (PMC10317219; doi:10.1371/journal.pone.0286069)
Supplement: S8 Table — (DOCX) [file pone.0286069.s008.docx]

**Supplementary Material**

## Supplementary Tables

**S8 Table. The reads number of the fungi in the five samples based on the *ITS2* sequences.**

| Genus | HSZY056 | HSZY143 | HSZY144 | HSZY162 | HSZY174 |
| --- | --- | --- | --- | --- | --- |
| *Alternaria* | 53 | 33 | 67 | 0 | 0 |
| *Aspergillus* | 52 | 53 | 97 | 13 | 35 |
| *Beauveria* | 18 | 0 | 0 | 0 | 0 |
| *Boeremia* | 23 | 29 | 14 | 0 | 0 |
| *Botrytis* | 52 | 33 | 36 | 0 | 0 |
| *Candida* | 0 | 0 | 13 | 0 | 66 |
| *Capsicum* | 0 | 0 | 10 | 0 | 0 |
| *Cephalotrichum* | 6 | 12 | 5 | 0 | 0 |
| *Cladosporium* | 56 | 52 | 86 | 0 | 6 |
| *Clonostachys* | 10 | 0 | 10 | 0 | 0 |
| *Diaporthe* | 190 | 102 | 127 | 0 | 0 |
| *Diospyros* | 0 | 0 | 8 | 0 | 0 |
| *Diplodia* | 8 | 11 | 11 | 0 | 0 |
| *Dothiorella* | 12 | 0 | 20 | 0 | 0 |
| *Fusarium* | 175 | 55 | 142 | 0 | 0 |
| *Hanseniaspora* | 10 | 19 | 0 | 0 | 0 |
| *Mucor* | 20 | 0 | 10 | 0 | 0 |
| *Nakazawaea* | 0 | 0 | 0 | 0 | 10 |
| *Papulaspora* | 19 | 0 | 0 | 0 | 0 |
| *Penicillium* | 88 | 77 | 129 | 0 | 19 |
| *Pichia* | 0 | 0 | 0 | 0 | 15 |
| *Plectosphaerella* | 10 | 0 | 10 | 0 | 8 |
| *Pseudogymnoascus* | 6 | 0 | 8 | 0 | 0 |
| *Saccharomyces* | 0 | 0 | 0 | 76 | 166 |
| *Schwanniomyces* | 0 | 0 | 0 | 74 | 648 |
| *Stagonosporopsis* | 6 | 0 | 0 | 0 | 0 |
| *Trichothecium* | 7 | 8 | 8 | 0 | 0 |
| *Uncultured* | 23 | 7 | 33 | 0 | 6 |
| *Uromyces* | 19 | 0 | 0 | 0 | 0 |
| *Vishniacozyma* | 6 | 8 | 0 | 0 | 0 |
