# Supplementary figures and images for: Validation of the shotgun metabarcoding approach for comprehensively identifying herbal products containing plant, fungal, and animal ingredients
Source: PLoS One. 2023 Jul 3;18(7):e0286069. doi: 10.1371/journal.pone.0286069 (PMC10317219; doi:10.1371/journal.pone.0286069)

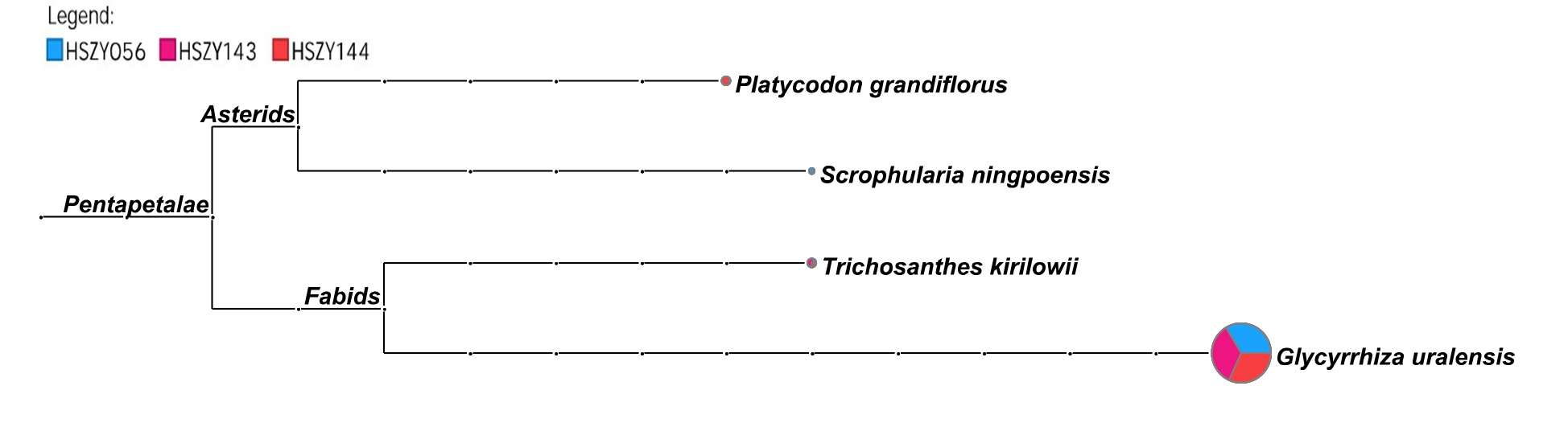

Supplement: S1 Fig — The node is drawn as a pie chart indicating the proportion of each species in the taxon for each sample. (TIF) [file pone.0286069.s009.tif]

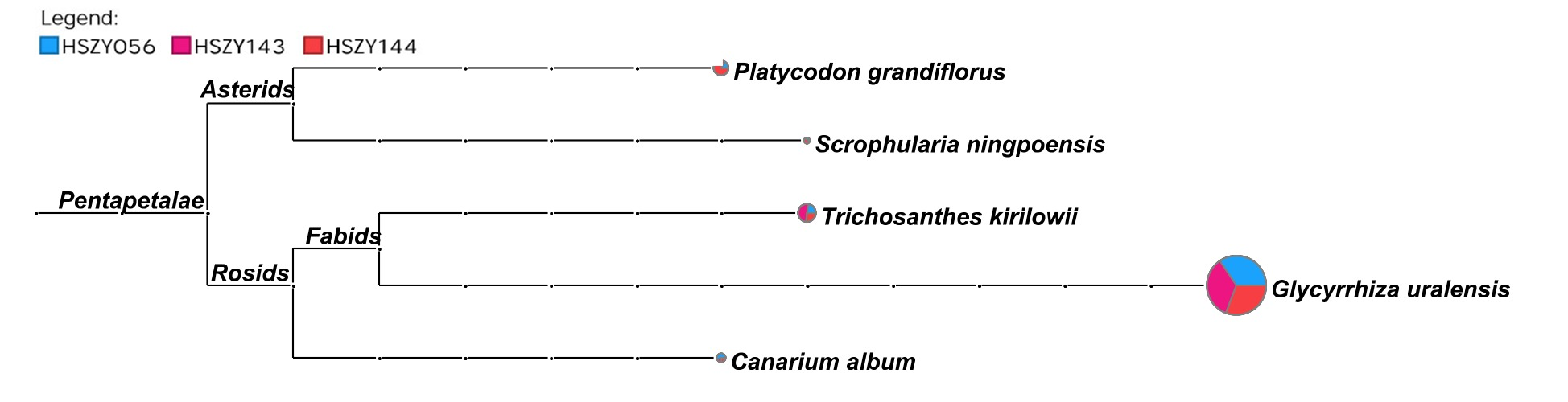

Supplement: S2 Fig — Each taxonomic the node is drawn as a pie chart indicating the proportion of each species in the taxon for each sample. (TIF) [file pone.0286069.s010.tif]

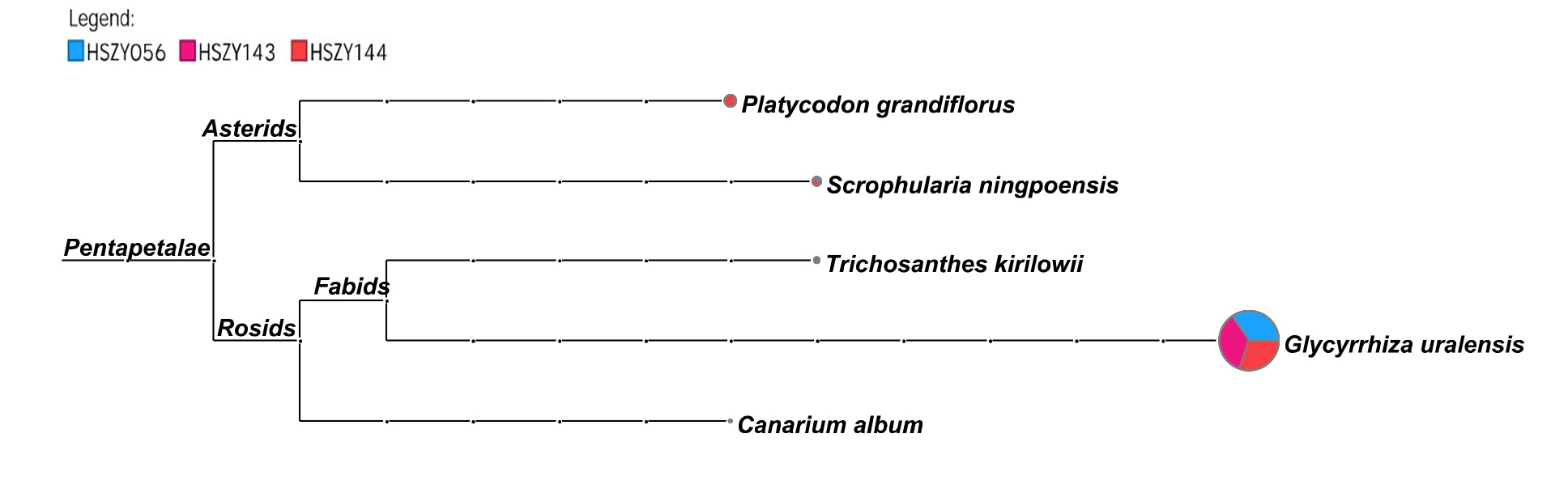

Supplement: S3 Fig — Each taxonomic the node is drawn as a pie chart indicating the proportion of each species in the taxon for each sample. (TIF) [file pone.0286069.s011.tif]

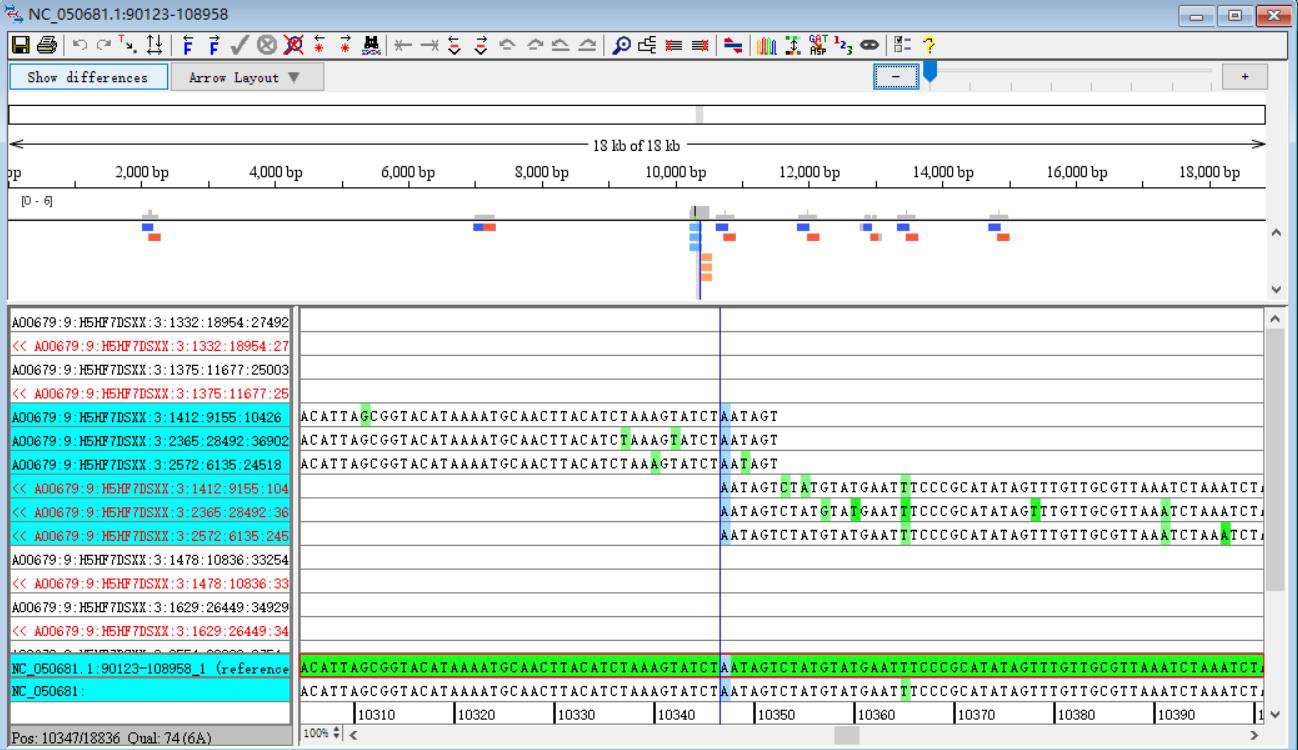

Supplement: S4 Fig — (TIF) [file pone.0286069.s012.tif]

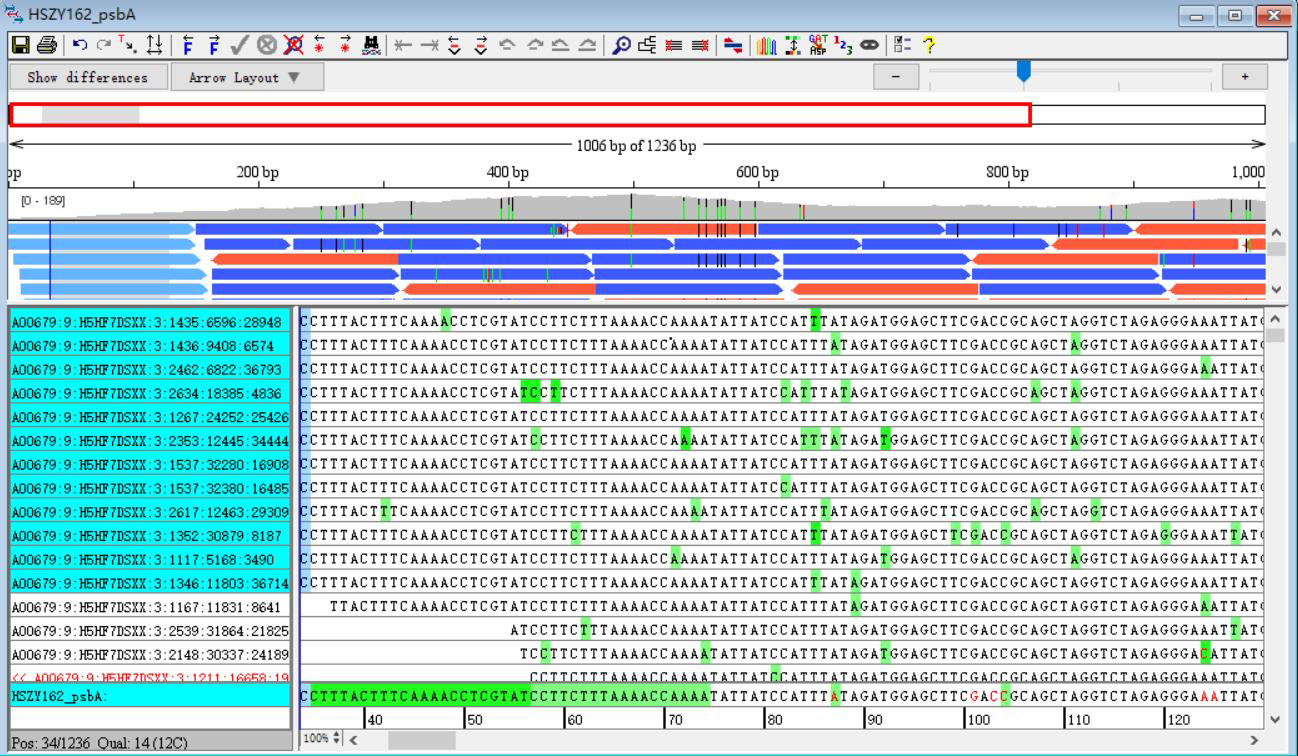

Supplement: S5 Fig — (TIF) [file pone.0286069.s013.tif]

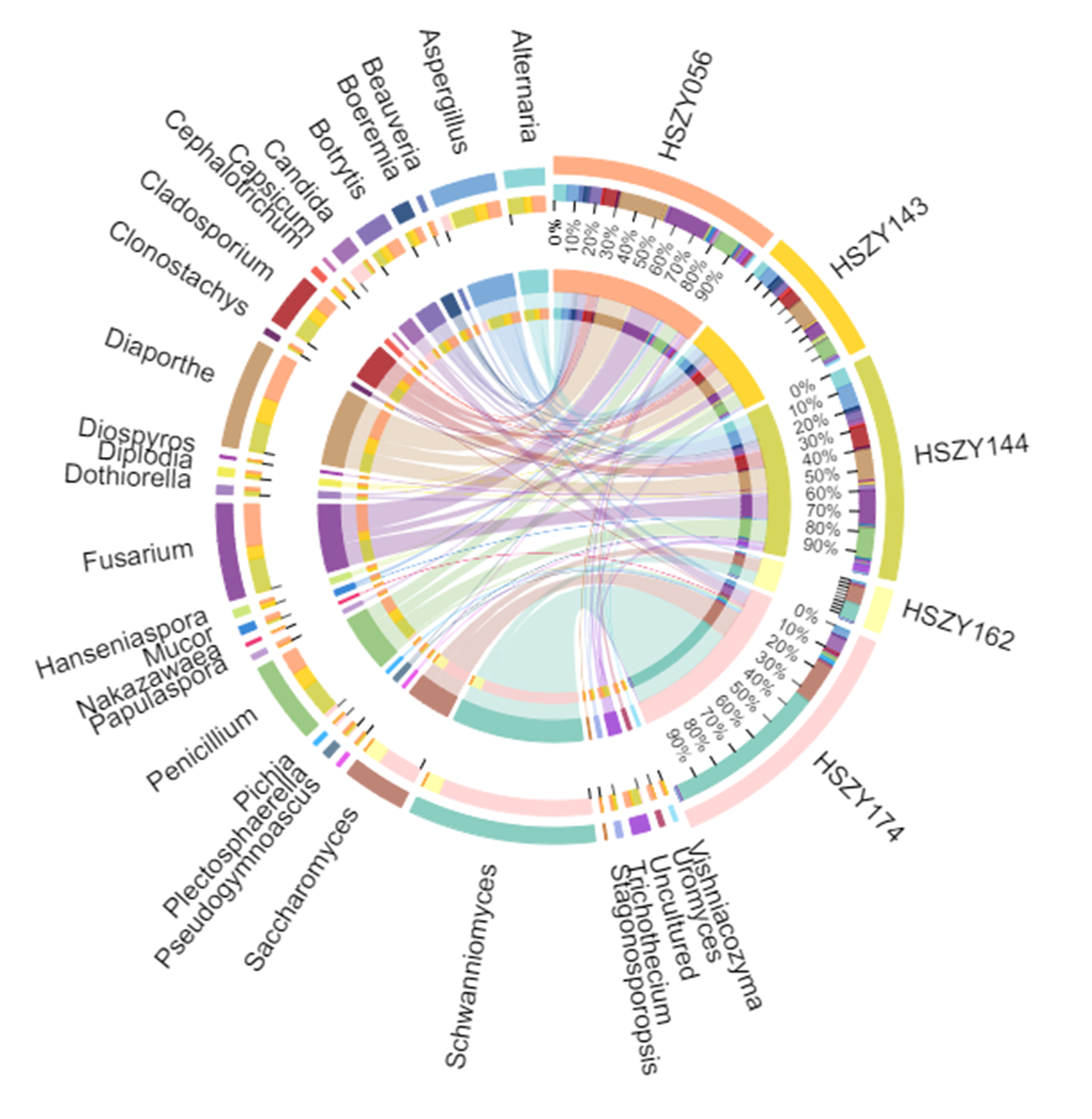

Supplement: S6 Fig — The data were visualized by Circos. The left half-circle indicates the distribution ratio of species in different samples at the genus level: the outer ribbon represents the species; the inner ribbon represents different groups, and the length represents the sample proportion of a particular genus. The right half-circle indicates the species composition in each sample: the color of the outer ribbon represents samples from different groups; the color of the inner ribbon represents the composition of different species in each sample, and the length of the ribbon represents the relative abundance of the corresponding species. (TIF) [file pone.0286069.s014.tif]
